# Bluetongue virus in carnivores: expanding the host range and implications for disease ecology

**DOI:** 10.1080/01652176.2025.2588740

**Published:** 2025-11-24

**Authors:** Aziz Ul-Rahman, Muhammad Zubair Shabbir, Jonas Johansson Wensman

**Affiliations:** a Faculty of Veterinary and Animal Sciences, MNS University of Agriculture, Multan, Pakistan; b Institute of Microbiology, University of Veterinary and Animal Sciences, Lahore, Pakistan; c Department of Animal Biosciences, Swedish University of Agricultural Sciences, Uppsala, Sweden; d Department of Microbiology, Swedish Veterinary Agency, Uppsala, Sweden

**Keywords:** Bluetongue, carnivore, host expansion, natural infection, experimental infection, epidemiological role

## Abstract

Bluetongue (BT), caused by the Bluetongue virus (BTV), is a vector-borne disease that primarily affects domestic and wild ruminants and is recognized globally for its significant impact on animal health, livestock productivity, and the economy. While traditionally considered as a disease confined to ruminants, recent evidence reveals that BTV has a broader host range, expanding to atypical species, including carnivores. This review consolidates current knowledge on natural and experimental BTV infection in atypical hosts, with particular emphasis on carnivores. The occurrence of acute or subacute infections, together with the detection of BTV-specific antibodies and viral RNA in carnivores such as dogs and lynx, points to a broader ecological interface. Expanding our understanding of BTV infection beyond classical ruminant hosts is essential for refining surveillance and control strategies, and for anticipating shifts in disease ecology under changing environmental and epidemiological conditions.

## 1. Introduction

Bluetongue (BT) is an arthropod-borne viral disease of domestic and wild ruminants that remains a significant threat to global livestock health (MacLachlan 2011; Sperlova and Zendulkova 2011). It is recognized as a notifiable disease under the World Organization for Animal Health (WOAH) Terrestrial Animal Health Code, and all confirmed cases must be reported to the organization. Historically, the disease was first observed in sheep in South Africa, where it caused devastating outbreaks among susceptible flocks. This disease was initially described using various names such as ‘Malarial Catarrhal Fever,’ and ‘Epizootic Catarrh of Sheep.’ The term ‘bluetongue’ (also spelled *blue tongue* or *blue-tongue*) was later adopted because of the striking clinical sign of a cyanotic swollen tongue observed in affected sheep in Africa. Early written descriptions of the disease from the late 19th and early 20th centuries (Spreull 1905), helped establish BT as a distinct disease entity and laid the groundwork for later virological studies (Afshar 1994).

Clinically, BT is characterized by fever, facial edema, nasal discharge, mucosal hemorrhages, lameness, and coronitis, with sheep being the most severely affected species (MacLachlan et al. 2009). In fatal cases, animals may develop a congested, swollen tongue that takes on a bluish discoloration due to vascular damage and hypoxia, which gave the disease its name. While sheep exhibit the most dramatic clinical signs, cattle, goats, and other ruminants usually experience milder or subclinical infections (Schwartz-Cornil et al. 2008). However, domestic cattle are now recognized as important reservoirs of BTV, as they can sustain prolonged viremia without obvious illness, thereby contributing to the maintenance and spread of the virus within susceptible populations (Qi et al. 2025). It is endemic in tropical and subtropical regions where competent *Culicoides* midges are present year-round, while seasonal incursions occur in temperate regions during the summer/warm months, coinciding with peak vector activity (MacLachlan 2011). However, recent outbreaks of BTV in Europe subsided with the onset of cooler weather, suggesting the potential for viral overwintering coinciding with the predicted start of vector–host transmission (Wilson et al. 2007). BTV outbreaks suggest that the virus may persist through infected vectors, subclinically infected cattle, or infected bovine fetuses, highlighting the complex epidemiology of BTV in temperate regions and underscoring the need to consider these mechanisms in understanding its distribution and re-emergence across Europe.

The disease reduces productivity through weight loss, reduced milk yield, abortions, infertility, and in severe cases, high mortality (Ganter 2014). Beyond direct animal health impacts, international trade restrictions on live animals and animal products from affected regions further amplify economic losses. BTV infection is estimated to cause an annual economic loss of USD 3 billion globally, including over USD 125 million in the USA.

The causative agent of BT is Bluetongue virus (BTV) (species *Orbivirus caerulinguae*) that belongs to the genus *Orbivirus* within the family *Reoviridae* (MacLachlan 2011; Matthijnssens et al. 2022). It is a non-enveloped virus with a genome composed of double-stranded (ds) RNA segments, which encode seven structural (VP1–VP7) and five additional non-structural proteins (NS1, NS2, NS3/NS3a, NS4 and NS5). The virus particle is spherical with an icosahedral symmetry and is made of an outer capsid (consisting of VP2 and VP5 proteins) and a double-layered core (VP3 forms the inner layer of the core and VP7 forms the outer layer of the core). VP2 and VP5 are responsible for host cell binding and viral entry in both mammalian hosts and insect vectors (Schwartz-Cornil et al. 2008). Precisely, VP2 plays the most significant role for serotype specificity by carrying the neutralizing epitopes (Stewart et al. 2015). The sequence variability of VP2 determines the virus’s antigenic diversity and forms the basis for the classification of BTV into at least 36 distinct serotypes (Ries et al. 2021). Wild ruminants have also been recognized as potential reservoirs, though their relative contribution to BTV circulation remains unclear. Several studies have documented BTV exposure among wildlife under natural and captive conditions (Rossi et al. 2014). Moreover, BTV-associated clinical disease and mortality have been reported in carnivores, including endangered species (Millán et al. 2009; Caballero-Gómez et al. 2024; Barros et al. 2025). However, the role of carnivores in the transmission cycle of BTV is still poorly understood. In this review, we summarize recent findings of natural and experimental BTV infections in carnivores, with a focus on vector-borne and interspecies transmission dynamics.

## 2. Historical emergence and global distribution

BTV was first reported in the Cape Province of South Africa in 1876, following the introduction of European Merino sheep for intensive farming. The disease name ‘Bluetongue’ is derived from the Afrikaans term ‘bloutong’ or ‘blaauwtong’, coined by local farmers to describe the cyanotic appearance of the tongue in affected animals (Spreull 1905). In 1933, the virus was first diagnosed in cattle (Bekker et al. 1934). Because of its clinical resemblance to foot-and-mouth disease, it was initially referred to as ‘pseudo-foot-and-mouth disease,’ ‘seerbeck,’ or ‘sore-mouth’ (Bekker et al. 1934). Initially confined to South Africa and other Sub-Saharan African regions, the virus gradually spread beyond the continent. The first major epidemic outside Africa occurred in Cyprus in 1943 (Gambles 1949), followed by outbreaks in Asia, the Middle East, North America, and Southern Europe during the mid-twentieth century (Walton 2004). The virus subsequently appeared in Australia (1977), South America (1980s), and has since become established in Central America, Asia, and the Mediterranean basin (MacLachlan 2004). Additional novel serotypes of BTV have also recently invaded Israel and Australia, countries in which ongoing surveillance over many years had confirmed an apparently stable cycle of annual BTV infection (Brenner et al. 2010; Firth et al. 2017). In Australia, multiple serotypes circulated between 1979 and 1986, with additional serotypes (BTV-2, −5, −7, −12, −16) emerging after 2007 and substantial transmission reported in New South Wales (Firth et al. 2017; White et al. 2019, 2021; Gestier et al. 2023). In the Middle East, Israel (2018–2020) reported nine serotypes, while Tunisia (2021–2022) and Cyprus (2022) confirmed new cases (Barua et al. 2024). In North America, BTV-2 strains have spread from the southeastern to the western United States, and novel strains have also emerged in the country through reassortment events (MacLachlan et al. 2013; Gaudreault et al. 2014; Schirtzinger et al. 2018). Multiple serotypes are currently circulating in South and Central America and the Caribbean (Legisa et al. 2014; Legisa and Dus Santos 2021; Acevedo et al. 2024), although seroconversion has not always been preceded by reports of clinical outbreaks (Navarro-Mamani et al. 2025). Interpretation of the BTV status of other regions of the world is complicated by the lack of adequate surveillance in many areas, particularly throughout much of Africa, Asia and the Middle East. However, there are data of multiple serotypes circulating in Zambia, India, Pakistan, Nepal, China and Iran in more recent years (Chambaro et al. 2020; Haque et al. 2025).

Throughout much of the twentieth century, bluetongue outbreaks in Europe were sporadic and largely confined to Southern regions. However, since 1998, the virus has expanded across Southern and Mediterranean Europe, driven largely by climate change and the northward shift in *Culicoides* vector distribution (Wilson and Mellor 2009). A major wave of outbreaks occurred between 1998 and 2002, followed by the unexpected emergence of BTV in Northern Europe in 2006, beginning in the Netherlands and rapidly spreading across northwestern Europe (Carpenter et al. 2009; MacLachlan 2010). After this epidemic wave, the BTV re-emerged at different times and in multiple regions. From 2014 onward, outbreaks were recorded in the Mediterranean basin, particularly in Italy, Spain, and the Balkans, while Western Europe also experienced recurring activity from 2015 to 2024 (Nomikou et al. 2015; Sailleau et al. 2017; Savini et al. 2017). These events highlight the continued circulation and re-introduction of the BTV across Europe, even after earlier epidemics appeared to be under control. Most recently, in September 2023, an outbreak was reported in the Netherlands, with rapid spread across Northern Europe (Holwerda et al. 2024). By the end of 2024, the virus had been detected in several additional European countries (Barua et al. 2024).

## 3. Virus transmission

Although over 1,000 species of *Culicoides* have been identified, fewer than 20 are considered competent vectors for BTV transmission (Carpenter et al. 2009; McGregor et al. 2022). The global distribution of BTV is largely determined by the ecological range of these vector species, which thrive in both tropical and temperate climates. The most common transmission route to ruminants is through the bite of infected hematophagous midges (MacLachlan 2011). Although *Culicoides* midges are weak fliers and rarely travel long distances on their own (Mellor et al. 2000), windborne dispersal of infected midges can enable long-distance spread, particularly across bodies of water, contributing to the incursion of BTV into previously uninfected regions, such as the southeastern United States from the Caribbean Basin (Johnson 2007; MacLachlan 2011; Burgin et al. 2013). Direct contact transmission, possibly *via* aerosols, has also been demonstrated among livestock (Batten et al. 2014).

In carnivores, BTV infection has mainly been associated with oral transmission following ingestion of infected tissues or meat rather than through *Culicoides* bites (Alexander et al. 1994; Jauniaux et al. 2008; Barros et al. 2025). In domestic dogs, the European lynx, and possibly other carnivorous species, sporadic cases have been linked to the consumption of infected placental tissues, aborted fetuses, or raw contaminated meat (Alexander et al. 1994; Barros et al. 2025). This mode of transmission might be especially pertinent in rural areas where traditional livestock management practices prevail. Herding dogs, for instance, may unintentionally expose themselves to high viral loads by consuming placental material or aborted fetuses after calving or lambing. This makes it possible for carnivores in these settings to become infected with BTV. Several infected dogs had documented contact with sheep, supporting this route of exposure (Holwerda 2023; Barros et al. 2025). Oral transmission has also been reported in lynxes following ingestion of infected meat (Millán et al. 2009). The notion of a natural infection in dogs was further supported by a recent instance in the Netherlands (2023) when a pregnant dog had clinical bluetongue during a BTV outbreak. However, the exact transmission route—whether oral ingestion or vector-borne—could not be definitively confirmed. Similarly, vector-borne BTV transmission to dogs has been suspected in Morocco (Dubovi et al. 2013; Evermann 2013).

Besides vector and oral routes, BTV transmission has also been linked to contaminated biological materials. Products such as fetal bovine serum have been suggested as potential sources of BTV exposure in carnivores. Furthermore, contamination of veterinary vaccines with BTV has been documented or suspected on several occasions—BTV-28 was identified in a contaminated vaccine, while BTV-6, BTV-11, and BTV-14 detected in Europe were genetically closely related to live-attenuated vaccine strains, suggesting accidental introduction through vaccine use (Maan et al. 2010; van Rijn et al. 2008; EFSA Panel on Animal Health and Welfare (AHAW)) 2017; Dal Pozzo et al. 2019). Vertical (transplacental) transmission has been particularly noted for BTV-8, which re-emerged in France in 2015, and has been implicated in congenital neurological malformations in calves during the European BTV-3 outbreak that started in 2023 (Duc et al. 2025; Romnée et al. 2025; Swinson et al. 2025; Venjakob et al. 2025). Additionally, several BTV serotypes have been detected in aborted ruminant fetuses in Israel, indicating that vertical transmission is not restricted to a single serotype (Golender and Hoffmann 2024).

## 4. Clinical manifestation of BTV

Bluetongue virus infection can manifest in peracute, acute, subacute, or chronic forms among a wide range of susceptible hosts, including domestic and wild ruminants as well as carnivores (MacLachlan et al. 2009; Falconi et al. 2011; Payan-Carreira and Simões 2025). In domestic ruminants, particularly sheep and cattle, the disease is characterized by high fever (up to 42 °C), increased respiratory rates, and hyperemia with swelling of the buccal, nasal, and ocular mucosa. Affected animals exhibit excessive salivation, nasal discharge, and cyanosis or ulceration of the tongue and oral mucosa. Acute cases in sheep are associated with high morbidity and mortality rates. Similar clinical presentations have been documented in domestic dogs and lynx species (Jauniaux et al. 2008; Millán et al. 2009; Caballero-Gómez et al. 2024; Hanekom et al. 2024; Barros et al. 2025). In dogs and lynx, clinical manifestation of BTV include frothy salivation, purulent ocular discharge, lip and nostril edema, and serous, mucopurulent, or hemorrhagic nasal discharge (Jauniaux et al. 2008; Dubovi et al. 2013; Barros et al. 2025). Notably, cyanotic, ulcerated tongues and oral epithelium necrosis have been observed in lynx cases (Millán et al. 2009; Caballero-Gómez et al. 2024). Systemic signs such as lethargy, anorexia, and vomiting are also common and may be linked to smooth muscle lesions in the pharyngeal region, predisposing affected animals to aspiration pneumonia and death (Jauniaux et al. 2008; Millán et al. 2009; Caballero-Gómez et al. 2024; Barros et al. 2025).

BTV-associated reproductive losses have also been documented in both ruminants and carnivores. In domestic ruminants, BTV infection during pregnancy can lead to fetal infection, congenital malformations, or abortion, with or without other clinical signs in the herd or flock, although specific data vary with virus serotype and host species (MacLachlan et al. 2009). In carnivores, particularly dogs, several reports describe abortion following natural or experimental infection (Evermann et al. 1994; Barros et al. 2025). In an experimental study, three of four pregnant dogs aborted following BTV infection (Brown et al. 1996). More recently, naturally infected pregnant dogs in Portugal also experienced abortion prior to death (Barros et al. 2025). Additional reports include three aborted canine fetuses (Dubovi et al. 2013), a single abortion case (Hanekom et al. 2024), and ten abortions in infected dogs (Evermann et al. 1994).

## 5. Gross pathology and histopathological changes

Natural and experimental BTV infections in sheep affect multiple organ systems, including the respiratory, digestive, reproductive, and cardiovascular systems. The disease is characterized by vascular endothelial injury, which leads to edema, hemorrhage, and tissue damage. A swollen, cyanotic (‘blue’) tongue is a classical but rare sign in severely affected sheep; however, it is absent in cattle, wild ruminants and carnivores (MacLachlan et al. 2008; Jahanroshan et al. 2023). Gross pathological lesions include hemorrhage and necrosis in the myocardium and at the base of the pulmonary artery. Additional findings comprise oral ulcers, edema of the lips and face, and coronitis (inflammation of the coronary band of the hooves) leading to lameness (MacLachlan et al. 2009). Hyperemia of the conjunctiva is also commonly observed in sheep. In dogs, pathological lesions include pulmonary edema with pleural effusion, hydrothorax, splenic necrosis, hepatic degeneration, renal glomerulopathy, and myocardial interstitial edema (Akita et al. 1994; Howerth et al. 1995; Brown et al. 1996; Dubovi et al. 2013; Caballero-Gómez et al. 2024; Hanekom et al. 2024; Barros et al. 2025). Histopathological findings included endothelial hypertrophy, myocardial interstitial edema, interstitial pneumonia with fibrinous exudation, glomerular enlargement and necrosis, diffuse interstitial pneumonia and lymphoid depletion in spleen and lymph nodes. Renal lesions included diffusely enlarged glomeruli, with glomerular capillary-lining cells showing pyknotic to karyorrhectic nuclei, and frequent fibrin thrombi. The presence of blood in the sinus areas of mesenteric lymph nodes confirmed the grossly observed hemorrhage (Brown et al. 1996). Clinically, affected dogs showed diffuse glomerulonephropathy, lymphocytic degenerative cardiomyopathy, centrilobular hepatocytic perivasculitis, and hepatic passive congestion (Howerth et al. 1995; Dubovi et al. 2013). In female dogs following abortion, edematous and necrotic uterine walls, accompanied by large volumes of foul-smelling black and green vaginal discharge, were observed (Dubovi et al. 2013).

Necropsy examination revealed congestion of abdominal organs, renal adhesions, hepatization of lung lobes with frothy fluid exudation, and generalized alveolar edema impairing respiration in dogs (Evermann et al. 1994; Barros et al. 2025). The BTV infection also proved lethal to lynx and caused deaths in Eurasian lynx (Evermann et al. 1994; Jauniaux et al. 2008). Necropsy examination revealed marked anemia, subcutaneous hematomas, petechial hemorrhages, pulmonary congestion with edema, emaciation, enlarged gelatinous lymph nodes, pneumonia, and widespread petechiae. Histologically, there was evidence of acute to subacute vasculitis affecting the musculature, myocardium, peritoneum, and lungs, characterized by edematous vessel walls and endothelial cell hypertrophy in lynx (Evermann et al. 1994; Jauniaux et al. 2008).

## 6. Evidence of BTV infection in carnivores

Multiple carnivore species have also been shown to develop antibodies against BTV (Table 1). Although infections in these species are often subclinical, fatal cases have been reported in European lynx (*Lynx lynx*) (Alexander et al. 1994; Caballero-Gómez et al. 2024) and domestic dogs (*Canis lupus familiaris*) (Akita et al. 1994; Brown et al. 1996; Dubovi et al. 2013; Hanekom et al. 2022; Barros et al. 2025). Serological surveys in several African countries have revealed high BTV antibody prevalence in both domestic and wild dogs, suggesting frequent exposure (Table 1). Experimental and field evidence further indicate that dogs and lynx are susceptible to infection and can mount detectable immune responses. While most infections remain asymptomatic, severe disease outcomes have been documented, particularly in pregnant dogs and European lynx (Table 1). In dogs, both natural and experimental infections have resulted in abortion and death, including an outbreak in the USA linked to BTV-11-contaminated vaccines and cases of naturally infected pregnant dogs in South Africa, Portugal, and the Netherlands (Evermann et al. 1994; Wilbur et al. 1994; Brown et al. 1996; Hanekom et al. 2024; Barros et al. 2025). Fatal BTV-associated disease has also been described in European lynx (Caballero-Gómez et al. 2024). Collectively, these findings demonstrate that although carnivores are not primary hosts, they can occasionally develop severe or fatal bluetongue disease.

**Table 1.** Evidence of bluetongue virus infection in carnivores.

Common name	Binomial name	Type of infection	Serotype(s)	Diagnostic method	Country	Clinical presentation	Reference
Black Bear	*Ursis americanus floridanus*	Natural	a	BTV antibodies by AGPT	USA	No	Dunbar et al. 1998
Cheetah	*Acinonyx jubatus*	Natural	BTV-8, BTV-13, BTV-17	BTV antibodies by cELISA, SNT	Tanzania	No	Alexander et al. 1994
Domestic Cat	*Felis catus*	Natural	BTV-6, BTV-9	BTV antibodies by cELISA, SNT	Kenya	No	Alexander et al. 1994
Domestic Dog	*Canis lupus familiaris*	Natural	BTV-11	Histopathological examination, BTV antibodies by cELISA, viral RNA by RT-PCR	USA	Yes‡	Akita et al. 1994
Domestic Dog	*Canis lupus familiaris*	Natural	a	Clinical description, histopathology, virus isolation, microscopy	USA	Yes	Evermann et al. 1994
Domestic Dog	*Canis lupus familiaris*	Natural	a	Virus isolation, electron microscope	USA	Yes	Wilbur et al. 1994
Domestic Dog	*Canis lupus familiaris*	Natural	BTV-3, BTV-4, BTV-8, BTV-10, BTV-13, BTV-14, BTV-17	BTV antibodies by cELISA, SNT	Botswana, Kenya	No	Alexander et al. 1994
Jackal	*Canis aureus*	Natural	BTV-3	BTV antibodies by cELISA, SNT	Botswana, Kenya	No	Alexander et al. 1994
Lion	*Panthera leo*	Natural	BTV-3, BTV-8, BTV-10, BTV-17	BTV antibodies by cELISA, SNT	Namibia, South Africa, Tanzania	No	Alexander et al. 1994
Spotted Genet	*Genetta genetta*	Natural	a	BTV antibodies by cELISA, SNT	Kenya	No	Alexander et al. 1994
Spotted Hyena	*Crocuta crocuta*	Natural	BTV-3, BTV-8, BTV-4, BTV-9, BTV-13, BTV-14, BTV-17	BTV antibodies by cELISA, SNT	Kenya	No	Alexander et al. 1994
Wild Dog	*Lycaon pictus*	Natural	BTV-2, BTV-3, BTV-4, BTV-8, BTV-9, BTV-10, BTV-13, BTV-17	BTV antibodies by cELISA, SNT	Botswana, Kenya, South Africa, Tanzania	No	Alexander et al. 1994
Domestic Dog	*Canis lupus familiaris*	Natural	BTV-11	BTV antibodies by AGID, SNT	USA	No	Howerth et al. 1995
Domestic Dog	*Canis lupus familiaris*	Experimental	a	BTV detection *in situ* hybridization	USA	Yes‡	Brown et al. 1996
Lion	*Panthera leo*	Natural	a	BTV antibodies by ELISA	Africa	No	Murray et al. 1999
Spotted Hyena	*Crocuta crocuta*	Natural	a	BTV antibodies by ELISA	Africa	No	Murray et al. 1999
Wild Dog	*Lycaon pictus*	Natural	a	BTV antibodies by ELISA	Africa	No	Murray et al. 1999
Eurasian Lynx	*Lynx lynx*	Natural	BTV-8	BTV antibodies by cELISA, viral RNA by RT-PCR	Belgium	Yes‡	Jauniaux et al. 2008
Iberian Lynx	*Lynx pardinus*	Natural	a	BTV antibodies by ELISA	Spain	Yes‡	Millán et al. 2009
Domestic Dog	*Canis lupus familiaris*	Natural	BTV-1	BTV antibodies by ELISA, SNT	Morocco	No	Oura and El Harrak 2011
Domestic Dog	*Canis lupus familiaris*	Natural	BTV-11	Viral RNA by RT-PCR, AGID, genome sequencing	USA	Yes	Dubovi et al. 2013
Domestic Dog	*Canis lupus familiaris*	Natural	a	Viral RNA by RT-PCR	South Africa	Yes	Hanekom et al. 2022
Domestic Dog	*Canis lupus familiaris*	Natural	a	BTV antibodies by cELISA	Turkey	Yes	Bayram and Gümüşova 2021
Eurasian Lynx	*Lynx lynx*	Natural	BTV-1, BTV-4	BTV antibodies by ELISA, VNT	Spain	Yes	Caballero‐Gómez et al. 2022
Iberian Wolf	*Canis lupus signatus*	Natural	BTV-1	BTV antibodies by ELISA, VNT	Spain	No	Caballero‐Gómez et al. 2022
Lion	*Panthera leo*	Natural	BTV-1	BTV antibodies by ELISA, VNT	Spain	No	Caballero‐Gómez et al. 2022
South American Sea Lion	*Otaria flavescens*	Natural	BTV-1	BTV antibodies by ELISA, VNT	Spain	No	Caballero‐Gómez et al. 2022
Fossa	*Cryptoprocta ferox*	Natural	a	BTV antibodies by ELISA	Spain	No	Caballero‐Gómez et al. 2022
Domestic Dog	*Canis lupus familiaris*	Natural	BTV-3	Viral RNA by RT-PCR	Netherlands	Yes	Holwerda 2023
Iberian Lynx	*Lynx pardinus*	Natural	a	BTV antibodies by cELISA, VNT	Spain	Yes‡	Caballero-Gómez et al. 2024
Domestic Dog	*Canis lupus familiaris*	Natural	a	qRT-PCR, cELISA, VNT	South Africa	Yes	Hanekom et al. 2024
Domestic Dog	*Canis lupus familiaris*	Natural	BTV-3	Viral RNA by RT-PCR, virus isolation	Portugal	Yes	Barros et al. 2025

ǂ Mortality, a: Not done/information not available/not applicable.

**Abbreviations:** BTV: Bluetongue Virus, AGPT: Agar Gel Precipitation Test, cELISA: Competitive Enzyme Linked Imunosorbent Assay, SNT: Serum Neutralization Test, VNT: Virus Neutralization Test, RT-PCR: Reverse Transcriptase Polymerase Chain Reaction, USA: United States of America.

**Note:** The species names are arranged in chronological order based on publication year from earlier to latest report.

## 7. Species conservation implications of BTV: Emerging threat or theoretical concern?

Endangered and vulnerable wildlife species often inhabit fragmented ecosystems and exist in small, genetically homogeneous populations, which may increase their vulnerability to emerging infectious diseases (Ul-Rahman et al. 2018, 2024; Rahman et al. 2019, 2020a, 2020b). Due to the complex interplay of vectors, reservoir hosts and the broad host susceptibility of BTV, a major challenge lies in identifying the ecological and epidemiological conditions that favor the viral emergence and spread before outbreaks occur, as well as in devising strategies to mitigate or prevent such epidemics. Although direct experimental evidence of BTV infection dynamics in carnivores is limited, the detection of BTV-specific antibodies and viral RNA in species such as dogs, lynx, hyenas, and bears (Table 1) suggests that spillover events may occur under certain ecological conditions (Brown et al. 1996; Millán et al. 2009; Caballero-Gómez et al. 2024; Barros et al. 2025). This raises important questions about the potential conservation implications of BTV for vulnerable and endangered carnivores, particularly in areas with limited surveillance and reporting capacity. The susceptibility of both small and large carnivores to BTV, if further confirmed, could pose a risk to already threatened populations. For instance, the Iberian lynx (*Lynx pardinus*), one of the world’s most endangered felids, might be particularly vulnerable due to its small and isolated population structure, with only an estimated 160–200 individuals surviving in the wild (Nowell and Jackson 1996). While no large-scale outbreaks have been documented, a hypothetical BTV introduction into captive breeding programs could have serious consequences, such as disrupting reproduction or reducing genetic diversity. Under such scenarios, disease-related mortality could exacerbate existing genetic bottlenecks, further limiting the adaptive potential of the species. These possibilities highlight the need for targeted surveillance, experimental studies, and disease-risk assessments to clarify the true impact of BTV on endangered carnivore populations.

## 8. Current knowledge gaps and future perspectives

The potential role of carnivores as reservoirs in the persistence and transmission of BTV to susceptible ruminant populations remains largely underexplored. Investigations confirming natural BTV infections in carnivores are scarce, with only a handful of case studies providing sufficient data to draw definitive conclusions regarding their susceptibility and epidemiological relevance. However, it is still uncertain whether carnivores act as amplifying hosts capable of maintaining the virus circulation or merely act as incidental hosts with negligible impact on transmission dynamics. Carnivores that consume infected carcasses or BTV-contaminated material, such as aborted fetuses or placental tissues, may transiently harbor the virus within their tissues or gastrointestinal tract. It is imperative to examine whether such animals can transmit the virus to susceptible hosts or insect vectors, either directly or indirectly. To date, no evidence supports direct carnivore-to-carnivore transmission of BTV, and the relative contributions of vector-borne versus oral transmission routes are yet to be clarified.

The incidence of asymptomatic or subclinical BTV infections in carnivores also remains unknown, which could result in an underestimation of the burden of BTV in these hosts. Furthermore, the pathogenesis of BTV in carnivores is poorly understood, with critical gaps in knowledge regarding species-specific susceptibility, viral tropism, replication kinetics, and host immune responses. Current risk assessment frameworks for BTV highlight the role of climate change in expanding vector habitats and facilitating virus emergence in new geographic regions (Bibard et al. 2025). Yet, the potential for BTV dissemination *via* the natural movement of domestic and wild carnivores across endemic and non-endemic zones remains underappreciated. This concern is amplified by the scavenging behavior of carnivores and the demonstrated environmental stability of BTV.

The low frequency of confirmed infections in dogs and other carnivorous species poses challenges for recognition and diagnosis. Most veterinary diagnostic laboratories do not routinely test for BTV in non-ruminant hosts, and existing diagnostic protocols for carnivores are largely empirical and insufficiently validated. This diagnostic gap underscores the need for a dynamic and broad-spectrum approach, in which veterinarians continuously update differential diagnoses by integrating clinical presentations, laboratory findings, and disease outcomes. In suspected cases, prompt implementation of supportive and targeted therapeutic strategies is crucial to prevent clinical deterioration and stabilize affected animals. Accurate diagnosis in symptomatic carnivores ultimately requires a multidisciplinary framework that combines clinical evaluation, molecular diagnostics, and epidemiological context.

## 9. Concluding remarks

The emergence of BTV infections in carnivores represents a significant paradigm shift in the understanding of the host range and transmission dynamics of BTV. Evidence suggests that carnivores, once considered incidental hosts, may in fact play a more complex role in BTV ecology than previously recognized. Current evidence indicates that transmission in carnivores is more likely to occur through oral ingestion of infected tissues rather than *via* vector-mediated routes, particularly in ecosystems with limited vector activity. Higher seroprevalence among free-ranging carnivores reinforce this hypothesis and highlight the importance of investigating alternative, non-vectorial transmission pathways. Despite high antibody prevalence, clinical cases in large carnivore species remain rare, suggesting that they generally do not develop overt disease upon exposure. Given the severity of clinical BT, the importation of visibly infected dogs is unlikely. However, subclinically infected yet viremic dogs from high-prevalence areas may explain unexplained BTV outbreaks. If capable of transmission, such dogs could play a larger role in BTV spread than previously thought. The detection of BTV RNA in dogs residing near infected sheep, as well as the occurrence of clinical BT cases in canines during active outbreaks in ruminants, further emphasizes the possibility of shared transmission environments between carnivores and herbivores. However, it remains uncertain whether these infections represent epidemiological dead ends or contribute, even marginally, to viral maintenance within affected ecosystems. While their role in BTV maintenance remains unclear, infections in carnivores, especially endangered species, warrant attention in conservation and surveillance programs. Incorporating carnivores into BTV monitoring programs, especially in endemic or outbreak-prone regions, could strengthen early detection efforts and provide critical insights into evolving epidemiological patterns under the One Health framework.

## Data Availability

There is no associated data.
